# Is There Any Relationship Between Primary Snoring and Carotid Intima–Media Thickness? A Cross-Sectional Study

**DOI:** 10.3390/jcm15010300

**Published:** 2025-12-30

**Authors:** Orhan Görgülü, Feride Fatma Görgülü

**Affiliations:** 1 Department of Otorhinolaryngology, Adana Health Practice and Research Center, University of Health Sciences, Adana 01500, Turkey; 2 Department of Radiology, Adana Health Practice and Research Center, University of Health Sciences, Adana 01500, Turkey; drferide@yahoo.com

**Keywords:** primary snoring, atherosclerosis, carotid intima–media thickness, vascular risk, vibration

## Abstract

**Background/Objectives**: Clinical observations linking primary snoring (PS) to early markers of vascular dysfunction suggest a possible contribution to subclinical atherosclerosis. This study aimed to evaluate carotid and femoral intima–media thickness (cIMT and fIMT) in patients with atherosclerosis with or without PS, and to identify potential determinants of increased cIMT. **Methods**: In this cross-sectional study, 140 patients with atherosclerosis enrolled. Participants were divided into two groups based on polysomnography results: patients with PS (*n* = 95) and patients without snoring (*n* = 45). Demographic data and anthropometric measurements were recorded for all patients. High-resolution B-mode ultrasound was used to measure cIMT and fIMT. Group comparisons, correlation analyses, and multiple linear regressions were performed to evaluate the relationship between IMT and anthropometric parameters. **Results**: Patients with PS had significantly higher cIMT than patients without PS (0.90 ± 0.15 mm vs. 0.65 ± 0.10 mm, *p* < 0.001, Cohen’s d = −1.83), whereas fIMT did not differ between groups (*p* = 0.185). Carotid IMT was positively correlated with age, body mass index (BMI), waist circumference, and neck circumference (all *p* < 0.001). Multivariate analysis identified age, waist circumference, and neck circumference as independent predictors of increased cIMT (adjusted R^2^ = 0.31, *p* < 0.001). Within the PS group, no significant difference was observed between cIMT and fIMT (*p* = 0.33). **Conclusions**: PS is strongly associated with increased carotid intima–media thickness in patients with atherosclerosis, independent of obstructive sleep apnea. The absence of a similar effect in the femoral artery supports the hypothesis that mechanical vibrations caused by snoring may cause local vascular damage in the carotid wall. These findings suggest that PS may represent an independent risk factor and an early marker of carotid atherosclerosis.

## 1. Introduction

Atherosclerosis is a chronic, progressive vascular disease characterized by lipid accumulation, inflammation, and the formation of a fibrous cap inside the arterial wall. It is the fundamental pathological substrate underlying serious cardiovascular events such as ischaemic stroke, myocardial infarction, and peripheral arterial disease, which remain the leading causes of death worldwide [1,2]. Although traditional risk factors such as high blood pressure, diabetes mellitus, dyslipidemia, obesity, and smoking are well-established [3,4], growing evidence suggests that non-traditional, potentially modifiable factors may also play a decisive role in atherogenesis [5,6,7].

Among these, sleep-related breathing disorders, particularly obstructive sleep apnea (OSA), have been shown to be important modifiers of cardiovascular risk. OSA is characterized by recurrent episodes of upper airway obstruction during sleep, resulting in intermittent hypoxemia, arousals, and excessive fluctuations in intrathoracic pressure [8]. These physiological disturbances activate the sympathetic nervous system, induce systemic inflammation and oxidative stress, and impair endothelial function, thereby accelerating the atherosclerotic process [9,10,11]. Large-scale clinical studies have demonstrated a strong link between OSA and high blood pressure, coronary heart disease, atrial fibrillation, heart failure, and stroke, as well as increased cardiovascular mortality [12,13,14].

However, the vascular effects of PS, defined by an apnoea–hypopnoea index (AHI) < 5 and the absence of clinical OSA, remain much less well understood [15]. PS, once considered a benign or socially embarrassing disorder, has recently attracted attention as a potential cardiovascular risk factor. Epidemiological data suggest that habitual snoring is widespread in the general population, but estimates vary due to the subjective nature of survey methods [16]. Recent studies using objective acoustic recordings over several nights have shown a strong link between snoring and high blood pressure, even after adjusting for OSA [17]. In addition, several clinical observations have associated PS with early markers of vascular dysfunction, including increased carotid artery intima–media thickness (cIMT), suggesting a contribution to subclinical atherosclerosis [18,19].

Experimental and mechanistic evidence also supports a plausible hypothesis of localized vascular injury. Snoring generates repetitive vibrations of the upper airway tissues that transmit mechanical energy to the nearby carotid arteries, which may lead to endothelial stress and inflammatory activation [20,21]. Furthermore, animal studies have shown that simulated snoring vibrations can directly alter endothelium-dependent vascular relaxation and reduce nitric oxide-mediated signal transmission in the carotid wall, even in the absence of structural damage [22]. These findings support the hypothesis that chronic vibrational stress may accelerate local atherogenesis through an “injury response mechanism” rather than through systemic hypoxia or metabolic dysregulation [23].

Despite these findings, significant gaps remain in our knowledge. Most studies conducted to date rely on subjective assessments or limited sleep studies covering only a single night, and few have examined whether the vascular effects of snoring are localized or systemic. Furthermore, the potential influence of PS, independent of OSA and traditional cardiovascular risk factors, remains insufficiently studied. Clarifying this distinction is essential, as it could lead to PS no longer being defined as a harmless phenomenon, but as a potential early indicator or contributing factor of localized vascular pathology. This study aimed to examine whether PS is associated with subclinical atherosclerosis through a localized vascular mechanism. Specifically, we compared the intima–media thickness (IMT) of the carotid artery and femoral artery in patients with atherosclerosis but without OSA. We hypothesized that PS is associated with increased carotid artery IMT, but not with femoral artery IMT, supporting the hypothesis that snoring-related vascular damage is primarily caused by local mechanical vibrations rather than systemic mechanisms.

## 2. Materials and Methods

### 2.1. Ethical Approval

This study was approved by the Clinical Research Ethics Committee of Adana City Hospital (Decision No: 128, Date: 25 October 2017, Meeting No: 8). All procedures were conducted in accordance with the ethical principles of the Declaration of Helsinki. Written informed consent was obtained from all participants before enrollment.

### 2.2. Study Design and Population

This cross-sectional observational study was conducted in the Otorhinolaryngology and Radiology outpatient clinics of Adana City Hospital. Patients aged 18–65 years with a diagnosis of atherosclerosis confirmed by clinical and imaging findings were screened for eligibility. A total of 140 consecutive eligible patients were enrolled. All participants underwent full-night polysomnography (PSG) in the hospital’s sleep laboratory.

According to PSG results, patients with an apnea–hypopnea index (AHI) < 5 were divided into two groups: those with objectively confirmed snoring were assigned to the PS group (*n* = 95), and those without snoring were assigned to the non-PS group (*n* = 45). Exclusion criteria included current or past smoking, being older than 65 years of age, a history of cerebrovascular accident, angina pectoris, chronic kidney disease, diabetes mellitus, pregnancy, or hormonal contraceptive use. Participants with incomplete PSG data or inadequate ultrasound image quality were also excluded.

### 2.3. Sleep Study and Snoring Assessment

Overnight PSG was performed in accordance with the American Academy of Sleep Medicine (AASM) standards. The parameters monitored included electroencephalography (EEG), electro-oculography (EOG), electromyography (EMG), electrocardiography (ECG), nasal airflow, thoracoabdominal movements, and pulse oximetry. Snoring was recorded using a built-in microphone and airflow sensor. Snoring phases were automatically detected and verified by a qualified sleep technician. The AHI was calculated as the number of apnea and hypopnea episodes per hour of sleep. Subjects with AHI < 5 and exhibiting signs of snoring were classified as primary snorers.

### 2.4. Data Collection

All participants underwent a detailed clinical examination. Demographic characteristics, medical history, and anthropometric measurements, including height, weight, neck circumference, and waist circumference, were obtained by trained staff using standardized techniques. Body mass index (BMI) was calculated as weight (kg) divided by height squared (m^2^).

### 2.5. Ultrasound Assessment of Intima–Media Thickness

Carotid and femoral IMT measurements were performed by a single experienced radiologist blinded to group allocation, using a high-resolution B-mode ultrasound system (Aplio 500, Toshiba Medical Systems, Tokyo, Japan) equipped with a 4.8–11 MHz linear-array transducer.

*Carotid IMT* (*cIMT*): Measurements were obtained from the far wall of the right common carotid artery (CCA), approximately 3 cm proximal to the carotid bulb in plaque-free segments. Three end-diastole measurements were averaged.

*Femoral IMT* (*fIMT*): Using an identical technique, three measurements were taken from the posterior wall of the right common femoral artery (CFA) proximal to the bifurcation, and the mean value was recorded as the subject’s fIMT.

### 2.6. Statistical Analysis

Statistical analyses were conducted using the R software package (version 4.4.2). The normality of data distribution was assessed using the Kolmogorov–Smirnov test. Continuous variables are presented as mean ± standard deviation (SD) for normally distributed data or median (interquartile range) for non-normally distributed data. In contrast, categorical variables are presented as numbers and percentages. Comparisons between participants with and without PS were made using the independent samples *t*-test for continuous variables and the chi-square test for categorical variables. The primary outcome variable was carotid intima–media thickness (cIMT), and the secondary outcome variable was femoral intima–media thickness (fIMT).

The effect size of the cIMT difference between the groups was calculated using Cohen’s d coefficient. Pearson or Spearman correlation analyses were used to evaluate the relationships between IMT values and continuous clinical variables, including age, BMI, waist circumference, and neck circumference.

Variables showing significant associations with cIMT in univariate analysis were entered into a multivariate linear regression model to identify independent predictors of increased cIMT. Additionally, within the PS group, paired *t*-tests were performed to compare cIMT and fIMT values to determine whether the vascular effect of PS was localized to the carotid artery. A two-tailed *p*-value < 0.05 was considered statistically significant.

## 3. Results

A total of 140 participants (95 with PS and 45 without PS) were included in the analysis. The two groups were similar in terms of age and sex distribution. However, individuals with PS had significantly higher BMI, waist circumference, and neck circumference than those without PS. The cIMT was significantly higher in the PS group, whereas fIMT did not differ significantly between groups (see Table 1).

### 3.1. Differences in Carotid and Femoral IMT Between Groups

**Figure 1:**
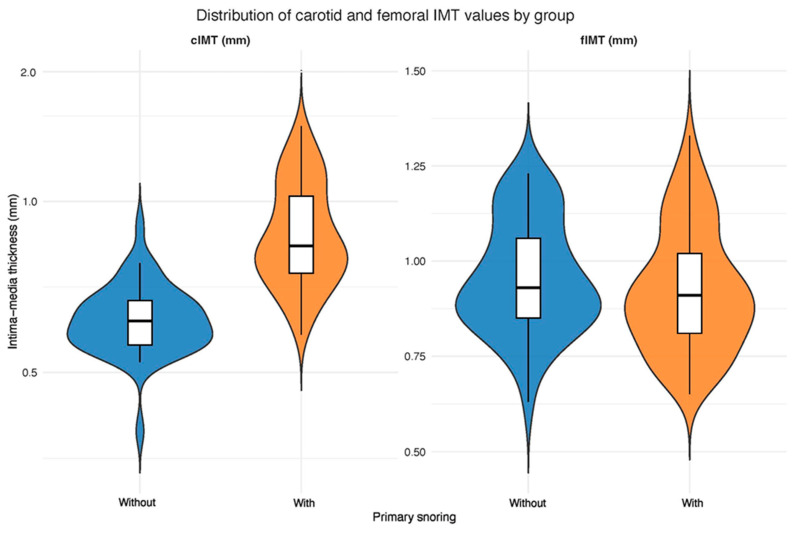
Violin plots illustrating the distribution of carotid (cIMT) and femoral (fIMT) intima–media thickness values in participants with and without PS. Each violin represents the kernel density of the data: the wider (bulged) sections indicate higher data density, whereas the narrower (flattened) sections represent lower density. Medians and interquartile ranges are marked within each violin.

The cIMT was significantly greater in individuals with PS compared to those without PS (t = −11.66, df = 123.3, *p* < 0.001). In contrast, femoral IMT (fIMT) did not differ significantly between the two groups (W = 2384.5, *p* = 0.185). The difference in cIMT between groups corresponded to a large effect size (Cohen’s d = −1.83, 95% CI [−2.25, −1.41]) (see Figure 1).

### 3.2. Associations Between Carotid IMT and Anthropometric Variables

**Figure 2:**
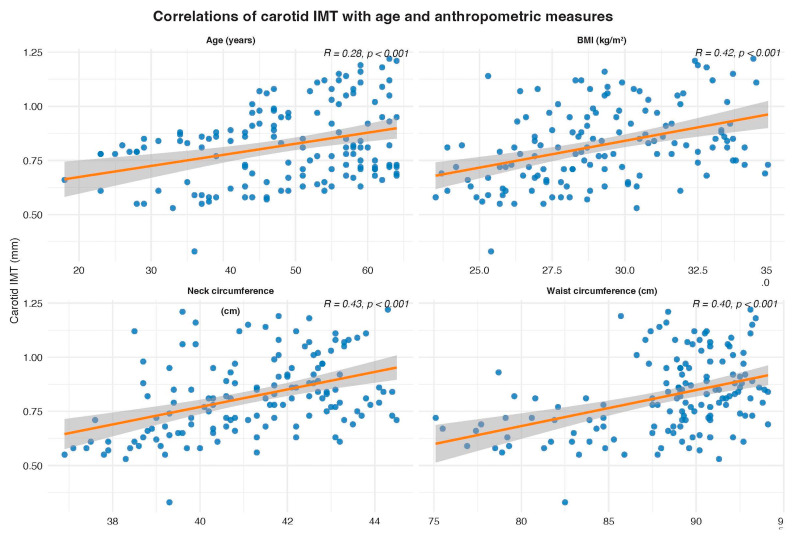
Scatter plots showing correlations between carotid IMT and age, BMI, waist circumference, and neck circumference. Correlation coefficients (R) and corresponding *p*-values are presented in each panel.

**Figure 3:**
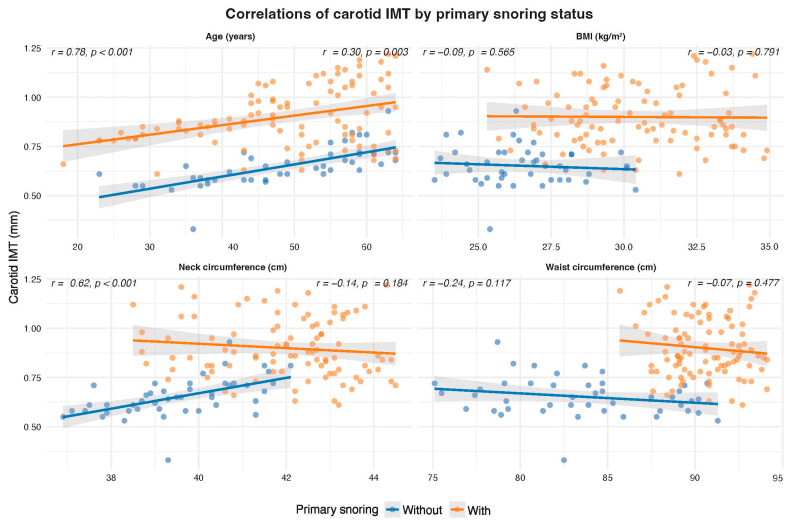
Correlations between carotid IMT and anthropometric variables by primary snoring status.

Spearman correlation analyses revealed a significant weak-to-moderate positive correlation between cIMT and several anthropometric and clinical parameters (*n* = 140) (see Figure 2). In the non-PS group, carotid IMT was strongly correlated with age (r = 0.78, *p* < 0.001) and neck circumference (r = 0.62, *p* < 0.001), but not with BMI or waist circumference. In contrast, among patients with PS, cIMT showed a weak but significant correlation with age only (r = 0.30, *p* = 0.003). In contrast, BMI, waist, and neck circumference were not significantly associated with cIMT (Figure 3).

### 3.3. Independent Predictors of Carotid IMT

A multiple linear regression model was constructed with cIMT (mm) as the dependent variable and age, BMI, waist circumference, neck circumference, and the presence of primary snoring as predictors (*n* = 140). The model significantly predicted cIMT values, accounting for approximately 53% of the variance (adjusted R^2^ = 0.53, *p* < 0.001). Age and the presence of primary snoring remained independent predictors of increased cIMT, while BMI, waist circumference, and neck circumference were not significant predictors in the adjusted model (see Table 2).

### 3.4. Comparison of Carotid and Femoral IMT Within the Primary Snoring Group

**Figure 4:**
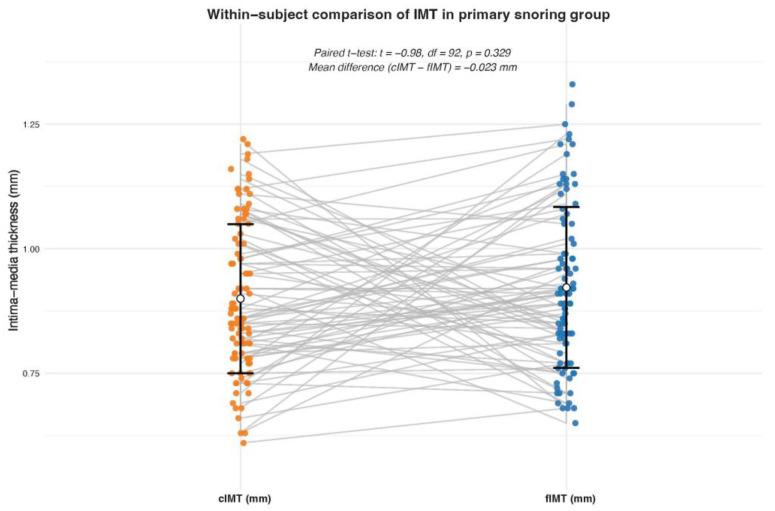
Within-subject comparison of IMT in the primary snoring group. Scatter plot comparing cIMT and fIMT intima–media thickness in individuals with primary snoring.

A paired *t*-test was performed within the PS group to compare cIMT and fIMT values. No significant difference was found between cIMT and fIMT values (*p* = 0.33) (see Figure 4).

## 4. Discussion

This study evaluated the influence of PS on carotid and femoral IMT and on clinical and anthropometric factors associated with carotid wall changes. Individuals with PS exhibited significantly greater carotid IMT compared with non-snorers, whereas femoral IMT values were similar between the two groups. Additionally, carotid artery thickening was positively correlated with age and the presence of primary snoring, while BMI, waist circumference, and neck circumference were not. These findings suggest that primary snoring, even in the absence of obstructive sleep apnea, may be associated with early vascular changes, particularly in the carotid arteries.

Despite no change in fIMT values in the snoring group, the significant increase observed in cIMT provides strong evidence that vascular changes in PS are regional rather than systemic. The location of thickening in the carotid arteries is consistent with the hypothesis that repetitive mechanical vibrations from snoring can directly damage the vessel wall. Experimental work by Cho and colleagues demonstrated that continuous oscillatory stimulation mimicking snoring impaired nitric oxide-mediated vasodilation in the carotid artery without significant endothelial damage [22]. Endothelial dysfunction is one of the earliest signs of atherogenesis [24] and triggers a series of inflammatory, thrombotic, and proliferative reactions that promote plaque formation [25].

Our findings regarding selective carotid thickening reflect this mechanism and suggest that chronic vibrational stress may induce localized endothelial damage that precedes clinical atherosclerosis.

The link between snoring and carotid artery damage has already been confirmed in human studies. Observational data have shown that habitual or loud snoring is a predictor of carotid atherosclerosis, independent of OSA severity and traditional cardiovascular risk factors [26]. Recent acoustic analyses have shown that snoring intensity and frequency are positively correlated with cIMT [23]. Our study confirms these correlations by directly comparing the carotid and femoral arteries in the same individuals. The absence of changes in cIMT indicates that the effect is not due to systemic inflammation or hypoxia, but to a localized vibration force transmitted through the neck tissues [27]. This pattern positions primary snoring as a clinical phenomenon rather than a milder form of OSA. On the contrary, the causative role of snoring in carotid atherosclerosis was not detected in a chronic simulated snoring rabbit model study [28].

Our study demonstrated a significant and independent association between primary snoring and increased carotid IMT, consistent with previous study [29]. This supports the hypothesis that snoring-related mechanical vibrations may promote localized carotid vascular remodeling. Although the snoring group had higher BMI, waist circumference, and neck circumference, multivariable linear regression analysis demonstrated that these anthropometric measures were not independently associated with cIMT. Femoral IMT did not differ between groups, despite similar exposure to systemic metabolic risk factors. This arterial-site specificity suggests that snoring-related mechanical vibrations may exert a localized effect on the carotid artery, beyond the contribution of generalized obesity. The large effect size observed (Cohen’s d = 1.83), together with the absence of femoral arterial involvement, further supports the hypothesis that snoring-related mechanical vibrations exert a regionally specific impact on the carotid artery. Although neck circumference was not independently associated with cIMT in the regression model, it may still serve as a dual indicator, reflecting both peripharyngeal fat deposition and exposure to mechanical vibrations during sleep [28,30]. While it is well known that central obesity promotes systemic atherosclerosis, our results show that the carotid artery, rather than the femoral artery, is disproportionately affected. This suggests that the mechanical effects of snoring may synergize with local obesity to increase endothelial stress in the neck region.

Growing evidence suggests that primary snoring should not be viewed solely as a social nuisance. Epidemiological studies have shown that objectively measured snoring, independent of OSA, is associated with uncontrolled hypertension [17]. According to individuals’ own statements, habitual snoring and excessive daytime sleepiness are factors that increase the risk of high blood pressure and cardiovascular disease, as is already known [31,32]. Our study adds a structural dimension to these findings: we suggest that vascular changes may already have begun, even in individuals who snore but have not been diagnosed with OSA. This outcome highlights the critical role of early intervention in preventing cardiovascular diseases. Measuring carotid intima–media thickness in habitual snorers may enable doctors to detect vascular lesions that have not yet manifested symptoms and take risk-reducing steps at a much earlier stage.

From a clinical perspective, if snoring contributes to regional damage to the carotid artery in the neck, the goal of treatment should be more than just ensuring a good night’s sleep or alleviating the bed partner’s discomfort. Theoretically, steps to reduce snoring severity, such as losing weight, correcting sleep posture, or using special oral appliances, may also alleviate pressure on the vessels. Indeed, Yaremchuk et al. emphasized that primary snoring treatment should not be viewed solely as a means to alleviate symptoms but also as a potential cardiovascular measure [33]. Lifestyle modification is a primary priority at this point: weight control, avoiding alcohol and sedatives, and finding the most suitable sleeping position are the first steps to take [34]. Additionally, splints that bring the jaw forward or intraoral appliances that stabilize the tongue may provide additional benefit in reducing vibrations in the airway [35,36].

### Study Strengths and Limitations

This study has several noteworthy strengths. Firstly, the accuracy of the classification is ensured because the snoring condition was objectively documented by polysomnography rather than relying on individuals’ self-reports. Secondly, the inclusion of individuals without sleep apnea (AHI < 5) in the study eliminated the confounding effect of sleep apnea on the results. Thirdly, the simultaneous examination of the carotid artery and the leg artery allowed for distinguishing whether vascular effects were regional or general, supporting the interpretation of mechanical damage. Finally, it is important that the effect size obtained (Cohen’s d = 1.83) reflects a clinically significant difference rather than a statistical coincidence.

However, the study has some limitations to consider. The cross-sectional design of the study does not allow us to establish a cause-and-effect relationship; repeated measurements over time (longitudinal data) are needed to determine whether snoring definitively triggers cIMT. The fact that participants were selected from patients examined for suspected vascular stiffness (atherosclerosis) may limit the extent to which the findings can be applied to the general population. Although known significant risk factors were controlled for, the possible effects of unmeasured factors such as dietary habits, physical activity level, or socioeconomic conditions on the results cannot be completely ruled out. While anthropometric measures differed between snorers and non-snorers, these variables were not independently associated with cIMT after adjustment; nevertheless, residual confounding cannot be entirely ruled out. Therefore, the findings should be interpreted with caution, and causality cannot be inferred.

Another limitation is that characteristics such as the duration, frequency, and noise level of snoring were not measured numerically. Previous studies have shown that objectively measured snoring duration is associated with arterial wall thickness, particularly in women [28]. Additionally, the fact that only the arteries on the right side were examined and that the consistency among the specialists performing the measurements was not reported are other limitations. Ultimately, the failure to measure biomarkers indicative of inflammation or intravascular health has prevented the direct verification of the proposed pathophysiological mechanisms. Finally, since a single radiologist performs IMT measurements, the lack of inter-observer or intra-observer repeatability assessment should be considered a limitation.

## 5. Conclusions

This study indicates that primary snoring, independent of obstructive sleep apnea, is associated with a significant increase in carotid intima–media thickness. The absence of changes in femoral intima–media thickness strongly suggests that this vascular effect is localized and most likely mediated by mechanical vibrations transmitted to the carotid artery during snoring. The absence of a similar change in carotid artery wall thickness provides strong evidence that this vascular effect is regional and most likely arises from mechanical vibrations generated during snoring and propagated to the carotid artery.

These results support the concept that primary snoring is not merely a harmless condition but may represent an early stage of vascular damage. From a clinical perspective, these findings highlight the importance of recognizing habitual snoring as a potential indicator of cardiovascular risk. Using carotid ultrasound to assess patients with snoring, particularly those with additional risk factors, may help detect subclinical atherosclerosis at a reversible stage before symptoms manifest. Future studies should focus on confirming this cause-and-effect relationship through long-term follow-up and intervention studies. Numerical analysis of characteristics such as the duration, severity, and frequency of snoring may help us understand the dose–response relationship in vascular remodeling.

## Figures and Tables

**Table 1:** Baseline demographic and clinical characteristics of the participants.

Variable	Without PS (*n* = 45)	With PS (*n* = 95)	*p*-Value
Age (years)	48.8 ± 11.1	48.5 ± 11.7	0.877
Sex (Male), *n* (%)	39 (86.7%)	85 (89.5%)	0.839
Body mass index (kg/m^2^)	26.6 ± 1.8	30.4 ± 2.4	**<0.001**
Waist circumference (cm)	83.7 (8.2)	90.6 (3.1)	**<0.001**
Neck circumference (cm)	39.5 (2.0)	42.3 (2.3)	**<0.001**
cIMT (mm)	0.652 ± 0.099	0.900 ± 0.150	**<0.001**
fIMT (mm)	0.930 (0.210)	0.910 (0.210)	0.185

**Table 2:** Multivariable linear regression analysis of predictors of carotid IMT (*n* = 140).

Variable	β (Estimate)	Std. Error	t	95% CI	*p*-Value
Intercept	1.027	0.401	2.56	0.23–1.82	0.011
Age	0.0056	0.0010	5.79	0.0037–0.0075	<0.001
BMI (kg/m^2^)	−0.0026	0.0057	−0.45	−0.0139–0.0087	0.654
Waist circumference (cm)	−0.0028	0.0042	−0.67	−0.0111–0.0055	0.502
Neck circumference (cm)	−0.0087	0.0074	−1.17	−0.0233–0.0060	0.245
Primary snoring (Reference “No”)	0.299	0.0369	8.11	0.226–0.372	<0.001

Adjusted R-squared: 0.53; F-statistic: 32.41 on 5 and 133 DF, *p*-value: <0.001.

## Data Availability

The data presented in this study are available on request from the corresponding author.

## References

[1] Bazoukis G., Loscalzo J., Hall J.L., Bollepalli S.C., Singh J.P., Armoundas A.A. (2025). Impact of Social Determinants of Health on Cardiovascular Disease. J. Am. Heart Assoc..

[2] Saba L., Cau R., Vergallo R., Kooi M.E., Staub D., Faa G., Congiu T., Ntaios G., Wasserman B.A., Benson J. (2025). Carotid artery atherosclerosis: Mechanisms of instability and clinical implications. Eur. Heart J..

[3] Ji X., Leng X.-Y., Dong Y., Ma Y.-H., Xu W., Cao X.-P., Hou X.-H., Dong Q., Tan L., Yu J.-T. (2019). Modifiable risk factors for carotid atherosclerosis: A meta-analysis and systematic review. Ann. Transl. Med..

[4] Torres G., de la Torre M.S., Pinilla L., Barbé F. (2024). Obstructive sleep apnea and cardiovascular risk. Clínica Investig. Arterioscler..

[5] Albertsen I.E., Bille J., Piazza G., Lip G.Y.H., Nielsen P.B. (2024). Cardiovascular Risk in Young Patients Diagnosed with Obstructive Sleep Apnea. J. Am. Heart Assoc..

[6] Bažadona D., Matovinović M., Skorić M.K., Grbavac H., Belančić A., Malojčić B. (2023). The Interconnection between Carotid Intima–Media Thickness and Obesity: Anthropometric, Clinical and Biochemical Correlations. Medicina.

[7] Thareja S., Mandapalli R., Shaik F., Pillai A.R., Palaniswamy G., Sahu S., Cherukuri S.P., Younas S. (2024). Impact of Obstructive Sleep Apnea on Cardiovascular Health: A Systematic Review. Cureus.

[8] Sateia M.J. (2014). International Classification of Sleep Disorders-Third Edition. Chest.

[9] Atkeson A., Jelic S. (2008). Mechanisms of endothelial dysfunction in obstructive sleep apnea. Vasc. Health Risk Manag..

[10] Kohler M., Stradling J.R. (2010). Mechanisms of vascular damage in obstructive sleep apnea. Nat. Rev. Cardiol..

[11] Peracaula M., Torres D., Poyatos P., Luque N., Rojas E., Obrador A., Orriols R., Tura-Ceide O. (2022). Endothelial Dysfunction and Cardiovascular Risk in Obstructive Sleep Apnea: A Review Article. Life.

[12] Bradicich M., Pengo M.F., Steier J., Schwarz E.I. (2025). Cardiovascular effects of obstructive sleep apnoea and effects of continuous positive airway pressure therapy: Evidence from different study models. ERJ Open Res..

[13] Mazzotti D.R., Waitman L.R., Miller J., Sundar K.M., Stewart N.H., Gozal D., Song X., Chandaka S., Anuforo K., Greater Plains Collaborative (2024). Positive Airway Pressure, Mortality, and Cardiovascular Risk in Older Adults with Sleep Apnea. JAMA Netw. Open.

[14] Nithitsutthibuta K., Sonsuwan N., Uthaikhup S., Kiatwattanacharoen S., Kunritt J., Pratanaphon S. (2025). Effects of obstructive sleep apnea on vascular structure and function in adults with obesity and type 2 diabetes: A comparative study. Cardiovasc. Endocrinol. Metab..

[15] Meira e Cruz M., Soca R., Kryger M. (2020). How much is too much after all? Primary snoring as a remaining unsolved issue. J. Clin. Sleep Med..

[16] Ohayon M.M., Guilleminault C., Priest R.G., Caulet M. (1997). Snoring and breathing pauses during sleep: Telephone interview survey of a United Kingdom population sample. BMJ.

[17] Lechat B., Naik G., Appleton S., Manners J., Scott H., Nguyen D.P., Escourrou P., Adams R., Catcheside P., Eckert D.J. (2024). Regular snoring is associated with uncontrolled hypertension. npj Digit. Med..

[18] Kim J., Pack A.I., Riegel B.J., Chirinos J.A., Hanlon A., Lee S.K., Shin C. (2017). Objective snoring time and carotid intima-media thickness in non-apneic female snorers. J. Sleep Res..

[19] Taylor C., Kline C.E., Rice T.B., Duan C., Newman A.B., Barinas-Mitchell E. (2021). Snoring severity is associated with carotid vascular remodeling in young adults with overweight and obesity. Sleep Health.

[20] Amatoury J., Howitt L., Wheatley J.R., Avolio A.P., Amis T.C. (2006). Snoring-related energy transmission to the carotid artery in rabbits. J. Appl. Physiol..

[21] Rice T.B., Strollo P.J. (2011). A nuisance or nemesis: The adverse effects of snoring. Sleep.

[22] Cho J.-G., Witting P.K., Verma M., Wu B.J., Shanu A., Kairaitis K., Amis T.C., Wheatley J.R. (2011). Tissue Vibration Induces Carotid Artery Endothelial Dysfunction: A Mechanism Linking Snoring and Carotid Atherosclerosis?. Sleep.

[23] Lee G.-S., Lee L.-A., Wang C.-Y., Chen N.-H., Fang T.-J., Huang C.-G., Cheng W.-N., Li H.-Y. (2016). The Frequency and Energy of Snoring Sounds Are Associated with Common Carotid Artery Intima-Media Thickness in Obstructive Sleep Apnea Patients. Sci. Rep..

[24] Szabóová E., Lisovszki A., Rajnič A., Kolarčik P., Szabó P., Molnár T., Dekanová L. (2024). Subclinical Atherosclerosis Progression in Low-Risk, Middle-Aged Adults: Carotid Leads Femoral in IMT Increase but Not in Plaque Formation. J. Cardiovasc. Dev. Dis..

[25] Gimbrone M.A., García-Cardeña G. (2016). Endothelial cell dysfunction and the pathobiology of atherosclerosis. Circ. Res..

[26] Lee S.A., Amis T.C., Byth K., Larcos G., Kairaitis K., Robinson T.D., Wheatley J.R. (2008). Heavy Snoring as a Cause of Carotid Artery Atherosclerosis. Sleep.

[27] Drager L.F., Bortolotto L.A., Lorenzi M.C., Figueiredo A.C., Krieger E.M., Lorenzi-Filho G. (2005). Early Signs of Atherosclerosis in Obstructive Sleep Apnea. Am. J. Respir. Crit. Care Med..

[28] Nam H., Yang H.-J., Kim Y.-A., Kim H.C. (2013). Impact of Chronic Simulated Snoring on Carotid Atherosclerosis in Rabbits. J. Clin. Neurol..

[29] Deeb R., Judge P., Peterson E., Lin J.C., Yaremchuk K. (2014). Snoring and carotid artery intima-media thickness. Laryngoscope.

[30] Preis S.R., Massaro J.M., Hoffmann U., D’Agostino R.B., Levy D., Robins S.J., Fox C.S. (2010). Neck circumference as a novel measure of cardiometabolic risk: The Framingham Heart study. J. Clin. Endocrinol. Metab..

[31] Balagny P., Vidal-Petiot E., Kab S., Frija J., Steg P.G., Goldberg M., Zins M., D’oRtho M.-P., Wiernik E. (2024). Association of Snoring and Daytime Sleepiness with Subsequent Incident Hypertension: A Population-Based Cohort Study. Hypertension.

[32] Endeshaw Y., Rice T.B., Schwartz A.V., Stone K.L., Manini T.M., Satterfield S., Cummings S., Harris T., Pahor M. (2013). For the Health ABC Study Snoring, Daytime Sleepiness, and Incident Cardiovascular Disease in The Health, Aging, and Body Composition Study. Sleep.

[33] Yaremchuk K. (2020). Why and When to Treat Snoring. Otolaryngol. Clin. N. Am..

[34] Changsiripun C., Chirakalwasan N., Dias S., McDaid C. (2024). Management of primary snoring in adults: A scoping review examining interventions, outcomes and instruments used to assess clinical effects. Sleep Med. Rev..

[35] Ramar K., Dort L.C., Katz S.G., Lettieri C.J., Harrod C.G., Thomas S.M., Chervin R.D. (2015). Clinical practice guideline for the treatment of obstructive sleep apnea and snoring with oral appliance therapy: An update for 2015: An American Academy of Sleep Medicine and American Academy of Dental Sleep Medicine clinical practice guideline. J. Clin. Sleep Med..

[36] Tuomilehto H.P., Seppa J.M., Partinen M.M., Peltonen M., Gylling H., Tuomilehto J.O., Uusitupa M. (2009). Lifestyle intervention with weight reduction: First-line treatment in mild obstructive sleep apnea. Am. J. Respir. Crit. Care Med..

